# Artificial intelligence tools in clinical neuroradiology: essential medico-legal aspects

**DOI:** 10.1007/s00234-023-03152-7

**Published:** 2023-05-09

**Authors:** Dennis M. Hedderich, Christian Weisstanner, Sofie Van Cauter, Christian Federau, Myriam Edjlali, Alexander Radbruch, Sara Gerke, Sven Haller

**Affiliations:** 1 Department of Neuroradiology, Klinikum Rechts Der Isar, School of Medicine, Technical University of Munich, Ismaninger Str. 22, 81675 Munich, Germany; 2 Medical Radiological Institute, Bahnhofplatz 3, Zurich, 8001 Switzerland; 3 Department of Medical Imaging, Ziekenhuis Oost-Limburg Genk, Schiepse Bos 6, 3600 Genk, Belgium; 4 Department of Radiology, University Hospitals Leuven, Herestraat 49, 3000 Louvain, Belgium; 5 Division of Medicine and Life Sciences, Department Neurosciences, Hasselt University, Campus Diepenbeek, Agoralaan Building D, 3590 Diepenbeek, Belgium; 6 AI Medical, Goldhaldenstr 22a, CH-8702 Zollikon, Switzerland; 7 Faculty of Medicine, University of Zürich, Pestalozzistrasse 3. CH-8032, Zurich, Switzerland; 8 Department of Radiology, APHP, Hôpitaux Raymond-Poincaré & Ambroise Paré, DMU Smart Imaging, GH Université Paris-Saclay, U 1179 UVSQ/Paris-Saclay, Paris, France; 9 Laboratoire d’imagerie Biomédicale Multimodale (BioMaps), Université Paris-Saclay, CEA, CNRS, Inserm, Service Hopsitalier Frédéric Joliot, Orsay, France; 10 Department of Neuroradiology, University Hospital Bonn, Venusberg-Campus 1 53127, Bonn, Germany; 11 Penn State Dickinson Law, Carlisle, PA USA; 12 CIMC - Centre d’Imagerie Médicale de Cornavin, Place de Cornavin 18, 1201, Genève 1201, Geneva, Switzerland; 13 Department of Surgical Sciences, Radiology, Uppsala University, Uppsala, Sweden; 14 Faculty of Medicine, University of Geneva, Geneva, Switzerland; 15 Department of Radiology, Beijing Tiantan Hospital, Capital Medical University, Beijing, 100070 People’s Republic of China

**Keywords:** Artificial intelligence; Regulation; Clinical decision support; Privacy protection; Neuroradiology

## Abstract

Commercial software based on artificial intelligence (AI) is entering clinical practice in neuroradiology. Consequently, medico-legal aspects of using Software as a Medical Device (SaMD) become increasingly important. These medico-legal issues warrant an interdisciplinary approach and may affect the way we work in daily practice. In this article, we seek to address three major topics: medical malpractice liability, regulation of AI-based medical devices, and privacy protection in shared medical imaging data, thereby focusing on the legal frameworks of the European Union and the USA. As many of the presented concepts are very complex and, in part, remain yet unsolved, this article is not meant to be comprehensive but rather thought-provoking. The goal is to engage clinical neuroradiologists in the debate and equip them to actively shape these topics in the future.

## Medical malpractice liability

Key Points:

• Medical malpractice liability relating to the use of AI in clinical practice is still unsolved and legal frameworks are in flux.

• The use of interpretable AI models is preferable if they work just as well as black-box models.

• Any clinical AI tool must only be used according to the standard of care.

A large amount of AI-based medical software solutions has become available in the field of neuroradiology in the recent years [1]. The most commonly addressed use cases are segmentation and volume measurements of global or regional brain parenchyma or brain lesions, image enhancement, and clinical decision support (CDS), e.g., when it comes to the detection of intracranial hemorrhage or large vessel occlusions. Medical experts, lawyers, and regulatory authorities have heavily debated medico-legal implications resulting from the use of AI-based software tools in clinical practice in recent years [2–4]. This is particularly important for software that seeks to provide CDS. As state-of-the-art CDS tools will (at least in the foreseeable future) not work in a fully automated way, the ultimate responsibility for a diagnostic or therapeutic decision will likely remain with the physician, who has to validate the results of the CDS tool [3, 5, 6].

However, the question remains whether and how AI-based CDS tools used in clinical practice could potentially alter medical malpractice liability. No matter how sophisticated an AI algorithm is, its output will be wrong in some instances, which in turn may lead to patient harm and medical malpractice claims. The legal standards for medical malpractice liability differ from country to country but share common principles. In general, the physician needs to follow the standard of care, which is typically care provided by a competent physician who has a similar level of specialization and available resources [2, 7]. Against this backdrop, Price et al. described basically two scenarios where physician liability may result from using AI in clinical practice and patient injury occurs: first, if the AI makes a correct recommendation according to the standard of care but the physician rejects this recommendation, and second, if the physician follows an incorrect recommendation of the AI that lies outside the standard of care [2].

Two further considerations with implications for the use of AI-based CDS arise from the above-mentioned general principle for assessing medical malpractice liability. First, to be on the safe side, physicians should make sure to always follow the standard of care. In other words, AI currently functions more as a tool in clinical practice to confirm medical decisions rather than a tool that improves care by challenging the standard of care [2]. Second, we must acknowledge that the medical standard of care is constantly changing and depends on the available resources. In consequence, it might even become imperative for certain indications to use AI-based CDS tools and adopt their output in the future if their accuracy outperforms “conventional” decision-making at a certain point [3, 8].

When assessing the risk of medical malpractice liability, not only for AI-based CDS tools but for any software tool in general, it is also relevant to consider whether the tool itself was applied correctly—i.e., according to its intended use [1, 9, 10]. Deviation from the intended use may likely be considered outside of the standard of care and thus increase the risk of liability for a physician. In cases where an AI-based software tool is a medical device (see below), the intended use has to be stated in the process leading to marketing authorization, such as FDA clearance/approval in the USA and “Conformité Européenne” (CE) marking in the European Union [9].

For example, makers of Software as a Medical Device (SaMD) typically need to describe where their device fits in the diagnostic workflow, regardless of whether it is based on AI or not, by answering the following questions:

- Who are the intended users of the tool (e.g., physicians, nurses, or laymen)?
- Which patient group is the target (e.g., age range, asymptomatic population vs. patients, clinical presentation)?
- What input data are needed (e.g., technical aspects of radiological imaging, the possibility of active quality control)?
- What is the output of the SaMD and how is this supposed to be used in the diagnostic workflow (e.g., is it a fully automated diagnosis, a diagnostic recommendation, or worklist prioritization)?

The latter furthermore defines the degree of human supervision needed for correctly using the device. So far, fully autonomous AIs (which by default do not demand human experts to check and interact with their outputs) have not yet received marketing authorization in the field of neuroradiology in the EU or the USA. However, interesting advances towards this direction have recently been made for chest X-ray reading [11].

As stated above, it is of utmost importance to know the exact intended use of an AI tool to use it correctly in daily clinical practice. However, understanding the intended use is not always that easy, and even if the AI tool is used in accordance with its intended use, further problems exist.

First is the use of black-box solutions where the algorithm merely provides an output without any further insights how this output was reached. Consequently, it can be difficult (if not impossible) to assess the accuracy of the AI’s output (and to justify a medical decision in case of medical malpractice liability claims). Mitigation strategies for this problem include interpretability and explainability of AI tools, which are two distinct terms. Interpretable AI uses more transparent or “white-box” algorithms that can be understood by human experts. An example would be additive weights in a linear model or decision trees. Explainable AI on the other hand uses a second AI algorithm on top of a black-box model to give post hoc explanations for the algorithmic output. However, the currently available techniques may not be suitable to sufficiently explain black-box decisions on an individual patient level [12, 13]. Thus, interpretable AI tools should be favored in cases where they perform just as well as black-box algorithms. However, tradeoffs may be necessary in cases where a black-box performs better than an interpretable model. Still, using such noninterpretable tools in health care, including neuroradiology, should at least precede intensive testing of their safety and effectiveness, such as through clinical trials that are currently relatively rare in AI [14]. The need for rigorous external testing of medical AI has been repeatedly emphasized by large national and multinational societies, e.g., most recently by the French National Constitutional Ethics Council and the (CCNE) and the French National Digital Ethics Committee (CNPEN) [15].

Second is the lack of specific training: The vast majority of neuroradiologists in the current workforce have not been trained to use AI tools and to flag cases of malfunctioning. There are currently no best practice guidelines on who should use these tools, when, and how. Moreover, the development of training programs for the next generation of neuroradiologists is only in its infancy [16, 17].

In summary, although the technical backbone of AI is innovative and allows for new applications in neuroradiology, the risk of medical malpractice liability exists, and liability will be assessed according to current liability frameworks. However, these frameworks may change in the future. For example, the European Commission has recently proposed a new AI Liability Directive and a Directive on Liability for Defective Products [18, 19]. At the moment, however, neuroradiologists should use AI more as a confirmatory tool and according to the standard of care (since AI has not yet become part of the standard of care) [2, 20]. If AI tools are used according to the standard of care, physicians can likely protect themselves from medical malpractice liability if patient injury occurs. However, there is a great need to educate the current (and future) workforce of neuroradiologists to further enhance their understanding of AI and provide best practice guidance on the use of such tools.

## Regulation of AI-based medical devices

Key Points:

• Obtaining a CE mark (in the European Union) or FDA clearance/approval (in the United States) is a prerequisite for legally marketing AI-based medical devices and using them on patients in clinical routine.

• Marketing authorization for medical devices represents conformity assessment with regulatory standards and technical safety but does not necessarily demonstrate clinical usefulness.

• Close monitoring of AI-based tools used in clinical practice is important as part of postmarket surveillance.

Some AI tools used in clinical care are classified as medical devices. In order to be marketed and used on patients, any medical device, including software, has to comply with the local medical device regulations [9]. For example, the regulatory framework in the European Union leads to CE marking and, in the USA, to clearance or approval by the Food and Drug Administration (FDA). While some countries outside the EU and the USA accept either CE marking or FDA marketing authorization, additional frameworks exist in other countries, e.g., in Japan through the Pharmaceuticals and Medical Devices Agency (PMDA). Following Brexit, the UK’s Medicines and Healthcare Products Regulatory Agency (MHRA) has taken over responsibilities pertaining to the regulation of medical devices, which were previously handled by the EU. In consequence, the United Kingdom Conformity Assessment (UKCA) mark has been created for medical devices being marketed in Great Britain with a transition period until 30 June 2023 [21]. As regulatory clearance is a prerequisite for market entry, complying with different local regulatory frameworks is a complex and labor-intensive task for companies. Of note, Switzerland, for instance, recently decided to accept FDA-cleared or FDA-approved medical devices, thus lowering the regulatory burden for market entry [22].

### Regulatory framework in the european union: CE marking

Obtaining a CE mark is a prerequisite for marketing a medical device, including SaMD, in the European Union. While the CE mark is accepted in every Member State of the European Union, handling of SaMD with risk classes of IIa or higher (see below) is performed by private, decentralized institutions [9]. These so-called Notified Bodies have been accredited to do a conformity assessment leading to the issue of a CE mark. This process is laid out in the Medical Device Regulation (MDR) [23], which has been applied since 26th May 2021. A substantial amount of currently marketed medical devices have still obtained CE marking under the Medical Device Directive (MDD), the former legal act. The current transition period, where medical devices have to be recertified under MDR, has recently been extended until the end of 2027 for higher risk devices and until the end of 2028 for medium and lower risk devices [24].

The MDR deals with all sorts of medical devices, regardless of whether they are physical or software products. Most AI algorithms aiming for clinical use will constitute “stand-alone” SaMD (as opposed to software in a medical device, SiMD, which is used to run, for example, an ultrasound machine).

**Fig. 1:**
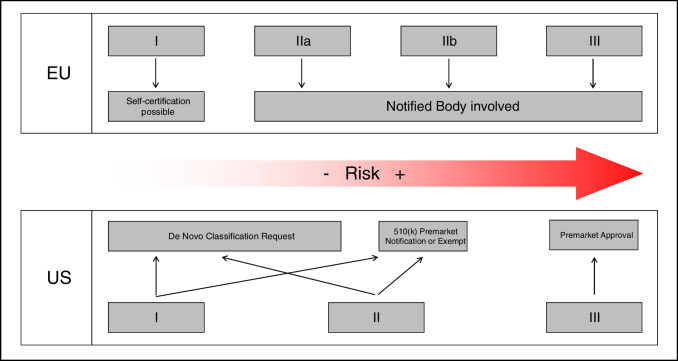
Overview of risk classes and associated pathways for obtaining marketing authorization in the European Union or the USA

The MDR recognizes four risk classes, spanning from I (minimal risk), IIa, IIb, to III (maximum risk), which determine the requirements under the MDR to place the device on the market (see Fig. 1). Any SaMD “provid[ing] information which is used to take decisions with diagnosis or therapeutic purposes” needs to be classified at least as risk class IIa [25]. Going further from here, it is important to consider the possible worst-case scenario (disregarding its probability). If one could think of a “serious deterioration of a person’s state of health or a surgical interventional,” the device will be classified as IIb; if “death or an irreversible deterioration of a person’s state of health” is possible, risk class III is assigned [25].

While under MDD, virtually all AI-based SaMD in neuroradiology belong to risk classes I and IIa; assignments of higher risk classes can be expected under MDR. For example, under MDD risk class I was commonly assigned for measurement tasks (e.g., brain volume measurements), which will happen less likely under the MDR framework due to the very broad definition of risk class IIa (see above). As all medical devices of risk class IIa need to be checked by a Notified Body, more frequent class IIa classifications will put pressure on the current regulatory infrastructure, which has probably led to the recent extension of the transition period. Without going into the details of necessary documents for CE marking, one should know that each developer seeking CE marking for a SaMD tool needs to write a supporting clinical evaluation of their tool and follow certain quality control measures. However, “clinical evaluation” does usually not comprise a rigorous clinical study performed with the tool but may also be based on retrospective data or data acquired for comparable devices [18, 23]. It is important for the clinical user to understand that CE marking certifies conformity with the EU MDR’s provisions but does not necessarily demonstrate its successful evaluation in a clinical environment.

### Regulatory framework in the USA: FDA clearance or approval

While medical device regulation in the European Union is carried out in a decentralized way through the frequent involvement of Notified Bodies, the FDA is the single agency in charge in the USA. Quite similar to the process in the European Union, a risk class is assigned based on the intended use definition of a SaMD, ranging from I (lowest risk) to III (highest risk). These risk classes also define the necessary steps to obtain FDA marketing authorization. In general, three different pathways exist (see Fig. 1) [9]:

Firstly, the so-called premarket approval or PMA needs to be obtained for high-risk devices (class III) and requires solid scientific evidence showing safety and effectiveness. Moreover, the so-called De Novo Classification Request is envisaged for low- to medium-risk (classed I and II) devices without any legally marketed predicate device, where general controls alone (class I) or general and special controls (class II) provide reasonable assurance of safety and effectiveness for the intended use. Lastly, the so-called 510(k) Premarket Notification/Clearance exists for new devices that are substantially equivalent to one or more “predicate” devices that are already legally on the market [14, 26].

Users should be able to assume that CE-marked and FDA-cleared SaMD are safe to use when applied according to their intended use. However, there is little evidence on how a SaMD performs on input data, which deviate from the data used during its development. This may refer to technical factors (e.g., slice thickness on input CT) as well as patient factors (using a tool designed for patients with Parkinsonian symptoms on asymptomatic patients). This underlines the need for close monitoring of a SaMD during its clinical applications, after it has received CE marking or FDA marketing authorization. So-called postmarket surveillance has gained more and more attention under the American and European frameworks and will probably become a cornerstone for monitoring the safe and responsible use of AI in clinical practice with the need for active participation of and guidance by neuroradiologists.

## Privacy protection in shared medical imaging data

Key Points:

• Privacy protection is critical for sharing medical imaging data, especially brain imaging.

• The way personal data or protected health information, respectively, is being handled is regulated by the GDPR in the European Union and HIPAA in the United States.

• Various software solutions exist to de-identify medical imaging data, including the removal of the face for 3D imaging data, but they need to be used with caution due to a risk of re-identification and potential corruption of medical imaging data.

• Federated learning may be a way to avoid sharing medical imaging data for training a machine learning algorithm.

As current AI algorithms need a large amount of training data during development, many hopes lie in large, aggregated imaging datasets. As imaging techniques like magnetic resonance imaging (MRI) have become more accessible and sharing large amounts of data has become feasible, several large-scale datasets have been collected and made available to the public [27, 28]. In addition, individual study datasets can be shared through repositories such as openneuro.org [29]. However, several important points have to be considered before sharing imaging data in neuroradiology.

First, who actually owns medical imaging data and is there a problem regarding ownership when it comes to data sharing? It has been stated that, in general, healthcare facilities in the USA have “‘ownership’ rights” over medical imaging data that they generate [30]. However, this ownership is usually not the only factor to consider since the patients are granted certain rights in their medical imaging, and their privacy needs to be protected when it is shared. Thus, privacy-protecting laws dominate the way medical imaging data can be shared [31]. Also, copyright questions are usually not problematic because medical images are often considered not copyrightable, as stated, for example, by the US Office Copyright Office [32]. Since this depends on national law, medical imaging data for use by researchers around the world is mostly distributed under a so-called CC-0 license to prevent copyright issues. A “CC-0 license” states that even if any copyright existed, it is being waived [33].

As stated above, not ownership but patient privacy is the crucial point when sharing medical imaging data. One factor that significantly determines how and if data sharing affects patient privacy is the degree of anonymization applied to the images, which lies within a spectrum. On the one hand, there is unprocessed data, as used in the hospital environment, where sensitive information such as name, date of birth, or address remains in the data and can be readily used to (re-)identify an individual. On the other hand, there is completely anonymized data without any possibility of retracing its origin. In between these two extremes of the spectrum, “de-identified” or “pseudonymized” data is commonly used for research and other purposes. For example, pseudonymization is done by removing all personal information. However, a key (i.e., a list of pseudonyms linking personal information with medical imaging data) exists that allows for swift re-identification of data by authorized persons.

The rules on data sharing depend on the country of origin. For example, in the USA and European Union, two major frameworks exist: the Health Insurance Portability and Accountability Act (HIPAA) [34] in the USA, whose Privacy Rule covers individually identifiable health information (called “protected health information” (PHI)), and the General Data Protection Regulation (GDPR) [35] in the European Union, which regulates personal data in general, including data concerning health as a special category of personal data.

While both frameworks seek to protect certain health information, there are some differences between the two. Under both GDPR and HIPAA, healthcare providers must usually provide access to PHI or personal data to patients upon request [36]. However, GDPR grants far broader rights to individuals to acquire and use their own personal data. Individuals may transfer, duplicate, or physically move their personal data files among different IT environments. They should receive the personal data “in a structured, commonly used and machine-readable format” and they “have the right to transmit those data to another controller without hindrance” [35, 36].

Another major difference between HIPAA and GDPR lies in how each law requires individuals to be informed about how their PHI or personal data is used, disclosed, and collected. The HIPAA Privacy Rule requires that covered entities, which include most healthcare providers, inform individuals about uses or disclosures of PHI through a Notice of Privacy Practices in plain language [37]. For example, a description of the circumstances under which healthcare providers may use or disclose PHI without obtaining written authorization has to be enclosed [37]. The GDPR gives individuals a right to be informed immediately when personal data is collected or used, which means “at the time when personal data are obtained” [35, 38]. Among other things, individuals must be informed who the recipients of the data are; and whether there is intent to transfer that data to third countries [35]. In contrast, if the personal data is not gained directly from individuals, the information must usually be given to them within a reasonable period of time [35, 38]. Individuals have the right to be informed in a transparent, precise, and easily accessible and comprehensible form [35, 38]. The GDPR grants individuals a much broader right to control how their personal data is collected, used, and disclosed than HIPAA does for PHI.

Medical imaging data obtained from patient populations needs associated information about the patient’s medical condition (e.g., symptom severity, environmental factors, genetic predisposition, to name a few) to be used effectively for SaMD as a diagnostic support tool. This information about a person’s health status is, of course, very sensitive and warrants the highest measures for privacy protection. It is important that neuroradiologists familiarize themselves with the applicable privacy laws when sharing patient data to protect their privacy adequately.

After providing a broad overview of HIPAA and the GDPR, we will now look at what kind of data is actually stored in medical imaging. Digital Imaging and Communications in Medicine (DICOM) objects and images are made up of both pixel data and metadata. Metadata includes information like the patient’s name, date of birth, and data about the particular scanner where the images were acquired. De-identification under HIPAA, for example, requires 18 specific identifiers that need to be eliminated in order to make a data set free of PHI [39]. Among those are the patient’s name, date and time, medical record numbers, social security numbers, device identifiers, full face, photographic and comparable images, and any other unique identifying number (e.g., the serial number of an implanted device such as a pacemaker) [34].

It can be very challenging to de-identify a dataset in case of so-called burned-in PHI. This could either be information like a project, a name, time, and date stamps in the image, which are common in ultrasound, fluoroscopic, radiographic images, and also secondary captures. These burned-in pixel data need to be “blacked out” (redacted) by replacing the pixel values using an image editor or dedicated tools. An overview about available software tools for de-identification or pseudonymization can be found in Table 1.

**Table 1:** Overview of software packages for de-identification or pseudonymization of medical imaging data

Name	Developer/vendor	Website	Cost	De-facing	Removal of burned-in information
DICOM Anonymizer	doRadiology.com	https://dicomanonymizer.com	Free	No	No
DicomCleaner	PixelMed Publishing	https://www.pixelmed.com/cleaner.html	Free	No	Yes
Horos	Horos Project	https://horosproject.org	10–500 USD	No	No
Niffler	Emory University Healthcare Innovations and Translationional Informatics Lab	https://emory-hiti.github.io/Niffler/	Free	No	No
RSNA MIRC Clinical Trials Processor	RSNA	https://www.rsna.org/research/imaging-research-tools	Free	No	Yes
PyDeface	Poldrack Lab at Stanford	https://github.com/poldracklab/pydeface	Free	Yes	No
mridefacer	Michael Hanke	https://github.com/mih/mridefacer	Free	Yes	No
mrideface	FreeSurfer	https://surfer.nmr.mgh.harvard.edu/fswiki/mri_deface	Free	Yes	No

Commonly used examples for software packages for de-identification of medical imaging data are given

Abbreviations: *DICOM*, Digital Imaging and Communications in Medicine; *RSNA*, Radiological Society of North America

A specific problem associated with 3D brain imaging is the possibility of reconstructing the face, which can then be processed by widely available face recognition software (e.g., for smartphone unlocking) [6]. Of particular note, an AI face recognition company was recently fined 20 million euros for several breaches under the GDPR [40]. There are a couple of different noncommercial approaches that allow the removal of facial features, such as PyDeface (https://github.com/poldracklab/), mridefacer (https://github.com/mridefacer/), and Freesurfer’s mrideface (https://surfer.nmr.mgh.harvard.edu/fswiki/mri_deface) [41]. However, they do not work in all cases and may affect the imaging properties of the brain itself. After de-facing with common software, a recent study has shown that 28 to 38% of scans still retained sufficient data for successful automated face matching [42]. Moreover, studies demonstrated that volumes and quality measurements are affected differently by de-facing methods. It is likely that this will have a significant impact on the reproducibility of experiments or performance if used by SaMD [42–44]. Therefore, it is always important to consider whether SaMD was trained on defaced or non-defaced images and which de-facing method was chosen if one enters new inference data [43].

As outlined in the paragraphs above, preparing medical imaging data for sharing without, for example, the restrictions of HIPAA (i.e., in de-identified form) can be quite complicated and prone to errors, so recently, other techniques have been proposed which avoid sharing data itself. One such concept is called “federated learning” [45]. It is a machine learning method designed for training models across a large number of decentralized edge devices or servers without the need for sharing training data. In a federated learning architecture, the decentralized edge device or server will first download the model and will improve it using local training data. After that, the federated learning method will push a small, focused update to the cloud, which is aggregated with updates from the edge devices or servers and distributed back. Throughout this process, the training data remains on the local servers and is not being shared itself. Only the updates to the algorithm weights are distributed to cloud environments. In summary, federated learning enables to build a machine learning model without sharing data, thus circumventing critical issues such as data privacy and security to a certain degree [46].

## Conclusion

In this article, we have provided an overview of the main medico-legal issues that pertain to using AI-based SaMD in clinical practice, namely medical malpractice liability, marketing authorization, and privacy protection of patient imaging data. Addressing these issues will be necessary for the safe and responsible adoption of AI in clinical practice and warrants active participation by neuroradiologists and other stakeholders, including legal experts.

## Data Availability

Not applicable (review article).

## Code availability

Not applicable (review article).
